# Myocardial fibrosis detected with gadolinium delayed enhancement in cardiac magnetic resonance imaging is related with arterioventricular coupling alterations in patients with acute myocarditis

**DOI:** 10.1186/1532-429X-17-S1-P345

**Published:** 2015-02-03

**Authors:** Christina Chrysohoou, Travis Henry, Arthur Stillman, Stamatios Lerakis

**Affiliations:** 1 Cardiac MRI, Emory University Hospital, Atlanta, GA, USA; 2 Cardiology, University of Athens, Athens, Greece

## Background

Delayed Gandolinium Myocardium Enhancement (DE) extent on Cardiac Magnetic Resonance (CMR) is significantly correlated to biomarkers of myocardial injury in patients with acute viral myocarditis, and is a significant independent predictor of adverse cardiovascular outcome. Arterial Venrticular Coupling (VAC) is related to the efficiency of mechanical energy transfer from the heart to the arteries, and it has been expressed by means of the ratio of effective arterial elastance (Ea) to end-systolic elastance (Ees), which is relatively independent of loading conditions. In this work we aimed to evaluate alterations in Ees, Ea and VAC indices in relation with DE in patients with acute myocarditis.

## Methods

61 patients (mean age 41± 17 years old, 59% male, 19% had hypertension, 8% coronary artery disease and 2% diabetes mellitus) with acute myocarditis were enrolled. All patients underwent echocardiographic evaluation, where cardiac chamber dimensions, stroke volumes (SV), bi-ventricular ejection fractions (EF), left ventricular isovolumic relaxation, isovolumic contraction and ejection times were measured. The estimation of VAC was according to the single-beat method; where Ea/Ees can be estimated non-invasively using echocardiographic and blood pressure measurements. DE in the left ventricle was evaluated with a CMR imaging. Both studies were conducted within a 24-hour period.

## Results

The mean ejection fraction was 43±18%; while 51% of the patients had preserved function. Those who exhibited DE had higher BMI (53±16 vs. 45±10 K/m^2^, p=0.07), lower VAC (0.97±0.16 vs. 1.12±0.49. p=0.08); lower Ees (1.94±0.95 vs. 2.28±1.08, p=0.1); lower Ea (1.87±1.12 vs. 2.38±1.08, p=0.09); higher left ventricular EF (46±16 vs. 40±19, p=0.08); higher left ventricular SV (62.5±21.6 vs.49±24.5, p=0.03); higher right ventricle EF (47 ±16 vs,39±21, p=0.08); while they showed no difference according to age, gender, hypertension, coronary disease diabetes mellitus. Multivariate regression analysis revealed that VAC was inversely related to DE (OR=0.014, CI 0-0.777, p=0.03), after adjustments for age, BMI, left ventricular chamber dimensions, coronary disease, hypertension, were made. When we stratified the analysis according to left ventricular EF, the relationship remained significant only in those with preserved left ventricular EF.

## Conclusions

It seems that in patients with acute myocarditis VAC shows lower values than we usually measure in patients with chronic heart failure. Especially in those with preserved EF, VAC is inversely related to the presence of fibrosis. It seems that in acute myocarditis with preserved EF, the healthy remained segments of the myocardium work towards optimization of ventricular mechanical efficiency instead of cardiac output maximization.

## Funding

None.

